# P-627. Geographic, Gender, and Racial Trends in Viral and Bacterial Pneumonia-Related Mortality in Adults with Co-existing Respiratory Failure, Aged 25 and Above in the United States, 1999-2020

**DOI:** 10.1093/ofid/ofaf695.840

**Published:** 2026-01-11

**Authors:** Saadia Ashraf, Hamza Asif, Kenneth Hannan, Zeeshan Ahmad

**Affiliations:** Khyber Teaching Hospital, Peshawar, Pakistan, Peshawar, North-West Frontier, Pakistan; University of Louisville Hospital, Louisville, KY; University of Louisville Hospital, Louisville, KY; University of Kansas Medical Center, Mission, Kansas

## Abstract

**Background:**

Pneumonia is a significant cause of morbidity and mortality. Despite its impact, long-term trends in viral and bacterial pneumonia-related mortality in adults with respiratory failure in the United States (U.S.) have not been thoroughly examined. This study analyzes temporal trends and geographical variations in viral and bacterial pneumonia-related mortality with respiratory failure in adults ≥ 25 years from 1999 to 2020.

**Figure:**
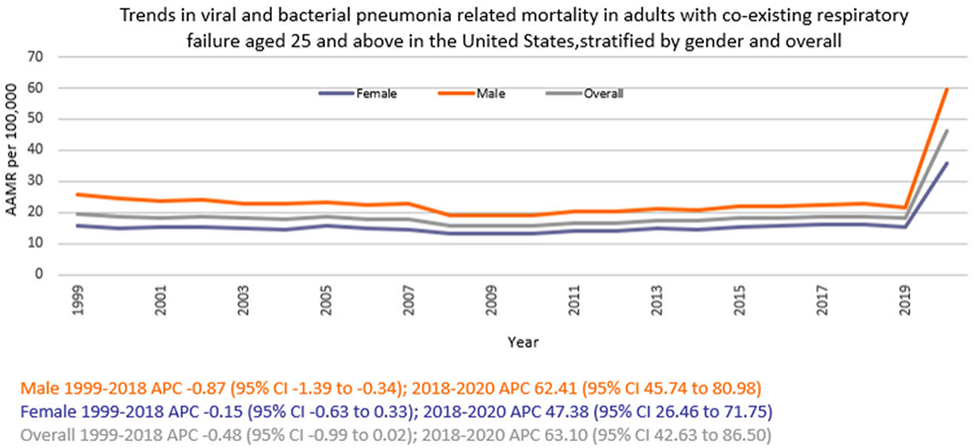

**Figure:**
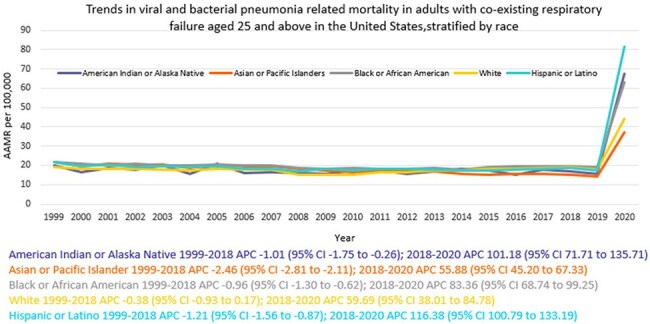

**Methods:**

We analyzed death certificate data from the CDC WONDER (Centers for Disease Control and Prevention Wide-Ranging Online Data for Epidemiologic Research) database between 1999 and 2020. Deaths due to pneumonia with co-existing respiratory failure in adults ≥ 25 years were examined, using the 2000 U.S. standard population for age standardization. Mortality rates were expressed as age-adjusted mortality rates (AAMR) per 100,000 population. Joinpoint regression was used to assess trends and calculate annual percentage change (APC), stratified by year, sex, race/ethnicity, census region, and states.

**Figure:**
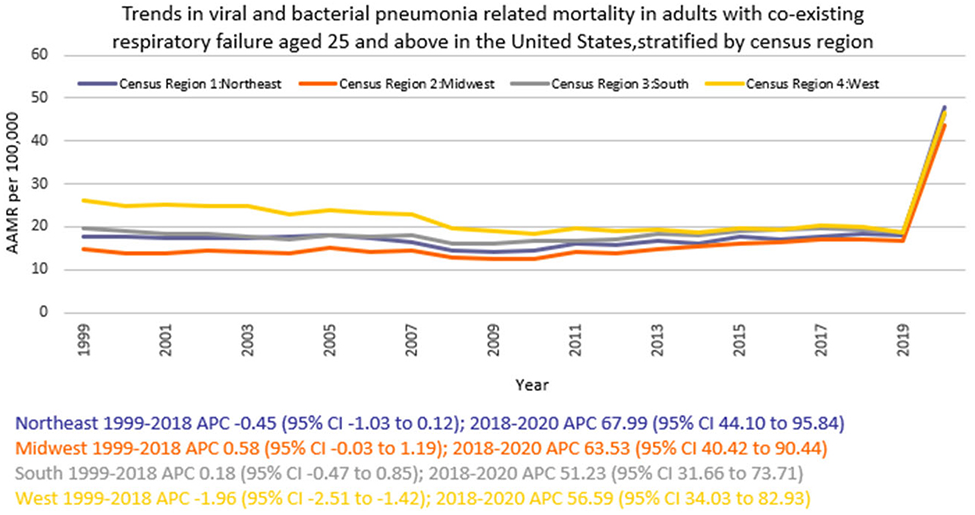

**Figure:**
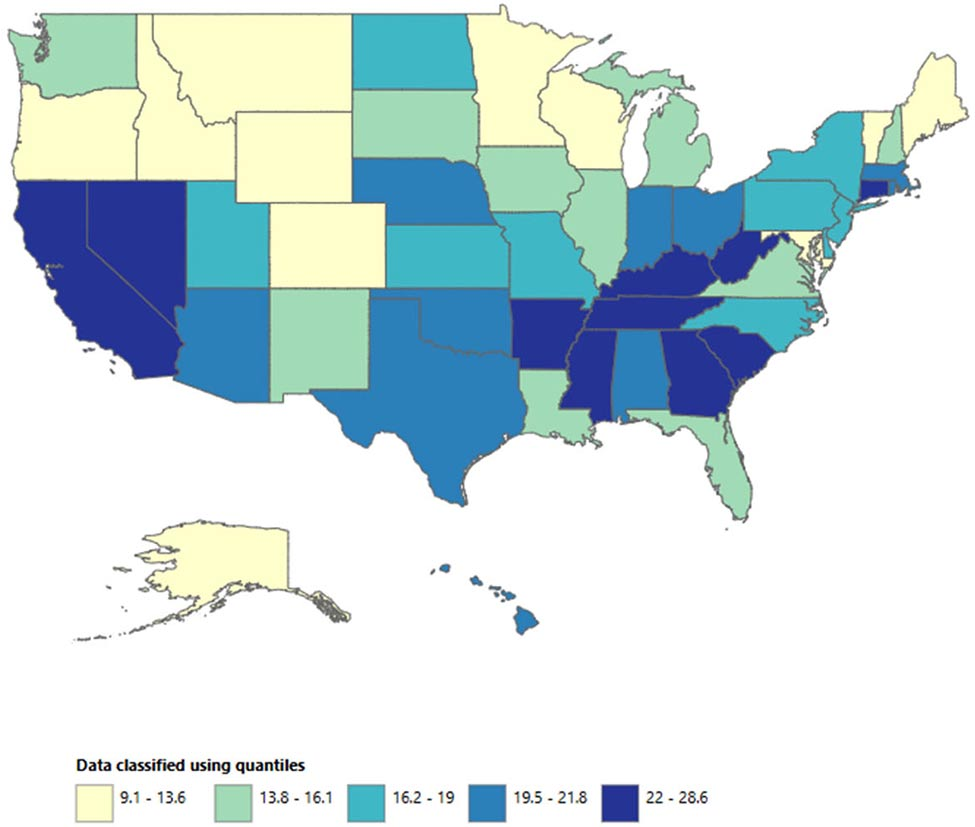

**Results:**

A total of 918207 viral and bacterial pneumonia-related deaths occurred between 1999 and 2020 in adults with co-existing respiratory failure. The AAMR decreased from 19.3 in 1999 to 18.8 in 2018 (APC -0.48; 95% CI: -0.99 to 0.02), and then increased to 46.1 by 2020 (APC 63.10; 95% CI: 42.63 to 86.50). Men consistently had higher AAMRs than women (24.1 vs. 15.9). Hispanics or Latino individuals had the highest AAMR (22.8), followed by Non-Hispanic (NH) Black or African American individuals (22), NH American Indian or Alaska Natives (20.6), NH Whites (19), and NH Asian or Pacific Islanders (18.5). Regional variations in AAMR were also significant, with the highest rates in the West (22.5), followed by the South (19.6), Northeast (18.4), and Midwest (16.3). Geographically, AAMRs ranged from 28.6 in California to 9.1 in Oregon.

**Conclusion:**

Overall, from 1999 to 2020, deaths due to viral and bacterial pneumonia in adults with respiratory failure initially decreased, followed by a sharp surge in the final years, with persistent disparities among men, Hispanic or Latinos, and residents of the West. These findings underscore the urgent need for targeted interventions and equitable healthcare access to mitigate these disparities and improve outcomes.

**Disclosures:**

All Authors: No reported disclosures

